# Usefulness of Antioxidants as Adjuvant Therapy for Septic Shock: A Randomized Clinical Trial

**DOI:** 10.3390/medicina56110619

**Published:** 2020-11-17

**Authors:** Alfredo Aisa-Alvarez, María Elena Soto, Verónica Guarner-Lans, Gilberto Camarena-Alejo, Juvenal Franco-Granillo, Enrique A. Martínez-Rodríguez, Ricardo Gamboa Ávila, Linaloe Manzano Pech, Israel Pérez-Torres

**Affiliations:** 1 Critical Care Department, American British Cowdray (ABC) Medical Center, I.A.P. ABC Sur 136 No. 116 Col. las Américas, Mexico City 01120, Mexico; alfredoaisaa@gmail.com (A.A.-A.); mesoto50@hotmail.com (M.E.S.); drcamarena@gmail.com (G.C.-A.); jfranco@abchospital.com (J.F.-G.); enrique_timc@hotmail.com (E.A.M.-R.); 2 Immunology Department Instituto Nacional de Cardiología Ignacio Chávez. Juan Badiano 1, Sección XVI, Tlalpan, Mexico City 14080, Mexico; 3 Physiology Department Instituto Nacional de Cardiología Ignacio Chávez. Juan Badiano 1, Sección XVI, Tlalpan, Mexico City 14080, Mexico; gualanv@yahoo.com (V.G.-L.); rgamboaa_2000@yahoo.com (R.G.Á.); 4 Cardiovascular Biomedicine Department Instituto Nacional de Cardiología Ignacio Chávez. Juan Badiano 1, Sección XVI, Tlalpan, Mexico City 14080, Mexico; loe_mana@hotmail.com

**Keywords:** shock septic, antioxidant therapy, oxidative stress, multiple organ failure

## Abstract

*Background and objectives:* Oxidative stress (OS) participates in the pathophysiology of septic shock, which leads to multiple organ failure (MOF), ischemia-reperfusion injury, and acute respiratory distress syndrome. Therefore, antioxidants have been proposed as therapy. Here, we evaluated the effect of antioxidant treatments in patients with septic shock with MOF and determined levels OS before and after treatment. This study was a randomized, controlled, triple-masked, and with parallel assignment clinical trial with a control group without treatment. *Materials and Methods:* It included 97 patients of either sex with septic shock. 5 treatments were used each in an independent group of 18 patients. Group 1 received vitamin C (Vit C), group 2 vitamin E (Vit E), group 3 n-acetylcysteine (NAC), group 4 melatonin (MT), and group 5 served as control. All antioxidants were administered orally or through a nasogastric tube for five days as an adjuvant to the standard therapy. *Results:* The results showed that all patients presented MOF due to sepsis upon admission and that the treatment decreased it (*p* = 0.007). The antioxidant treatment with NAC increased the total antioxidant capacity (*p* < 0.05). The patients that received Vit C had decreased levels of the nitrate and nitrite ratio (*p* < 0.01) and C-reactive protein levels (*p* = 0.04). Procalcitonin levels were reduced by Vit E (*p* = 0.04), NAC (*p* = 0.001), and MT (*p* = 0.04). Lipid-peroxidation was reduced in patients that received MT (*p* = 0.04). *Conclusions:* In conclusion, antioxidant therapy associated with standard therapy reduces MOF, OS, and inflammation in patients with septic shock.

## 1. Introduction

Damage caused by oxidative stress (OS) participates in the pathophysiology of serious diseases including multiple organ failure (MOF) due to sepsis. Sepsis is caused by bacteria, fungi, and viruses, or by a combination of them [1]. Sepsis and septic shock are the largest cause of mortality worldwide in intensive care units (ICU) [2] and MOF constitutes a high cost to health systems [3].

Studies in animal models and in patients with septic shock have shown an imbalance between the production of reactive oxygen (ROS) and nitrogen (RNS) species and antioxidant defenses [4]. ROS are generated by phagocytic cells, by the increased activity of enzymes such as NAD(P)H oxidase, xanthine oxidase and inducible nitric oxide (iNOS) and by increased inflammatory mediators through the activation of nuclear factor κB (NFκB) [5]. Mitochondrial damage caused by OS is a component of the pathophysiology of MOF secondary to sepsis [6].

Antioxidants such as N-acetylcysteine (NAC), melatonin (MT), vitamins (A, C and E), enzyme cofactors (selenium and zinc), and endogenous compounds (ubiquinone, α lipoic acid, bilirubin, albumin, ferritin, and quercetin) may inhibit ROS and RNS, counteracting their effects [7]. NAC has anti-inflammatory and antioxidant properties [8]. Its antioxidant capacity is due to the replenishment of glutathione (GSH) deposits and to the sequestration of ROS [9]. NAC improves hemodynamic variables, cardiac indexes, oxygenation and compliance of lung statics [10], hepatosplenic flow, and liver function in septic shock. Thus, NAC could decrease MOF [11] and reduce the levels of IL-8, soluble α receptor tumor necrosis factor p55 [12], IL-6, and ICAM-1 [13]. It reduces mechanical ventilation length, number of days in the ICU, and mortality [14].

Vitamin C (Vit C) can reduce the production of nitric oxide by the iNOS pathway and it may decrease vasoconstriction and loss of vascular permeability [15]. Decreased Vit C levels are related to the severity of MOF and mortality [16]. In some clinical studies, therapy with Vit C decreased sequential organ failure assessment (SOFA) scores, procalcitonin (PCT), C-reactive protein (CRP), and thrombomodulin, leading to a lower mortality rate [17]. Several studies have shown that vitamin E (Vit E) is an important lipophilic antioxidant in cell membranes, protecting them from lipid peroxidation (LPO) [18]. It has also been reported that the administration of Vit E in combination with simvastatin inactivates NAD(P)H oxidase, a source of ROS, in patients with sepsis that have decreased levels of Vit E and O_2_^−^ overproduction [19].

Melatonin (MT) lowers OS both in plasma and intracellular membranes due to its hydrophilic and lipophilic properties. MT possesses ROS sequestration properties, thus protecting cell membrane lipids, cytosol proteins, and nuclear and mitochondrial DNA [20].

There is a marked increase in ROS and a decrease in endogenous antioxidant defenses in critically ill patients with sepsis [21]. However, the usefulness of different antioxidants has not yet been evaluated through clinical randomized trials. Thus, the aim of this study was to evaluate the antioxidant effect of Vit C, Vit E, NAC, and MT in patients with septic shock determining the SOFA score and measuring antioxidant markers in plasma.

## 2. Material and Methods

### 2.1. Study Population

This was a controlled, randomized, and triple masked clinical trial that included 97 patients of either sex with septic shock. It was run in 2 ICUs in Mexico City. Patients were admitted to the ICU with a primary diagnosis of septic shock. Diagnostic criteria for septic shock were based on the Sepsis-3 consensus [22], and patients had to fulfill the criteria within a maximum of 24 h prior to enrollment. Data were collected upon admission to the ICU. In addition, other patients met selection criteria during their stay in intensive care, and they were then randomized. Patients had to have an acute increase of at least 2 points in the SOFA score [23], lactate level greater than 2 mmol/L, and they had to be dependent on a vasopressor for at least 2 h before the time of enrollment. Exclusion occurred when patients were younger than 18 years, when they were not able to grant an informed consent or refused to be included, if they were pregnant or breastfeeding or if they were under chronic use (last 6th months) or recent use of steroids, statins or antioxidants. Patients were also excluded if there was any contraindication for the use of Vit C, Vit E, NAC, or MT.

Ethical approval was obtained from the local ethics committee 24 the April of the 2018 (INcar PT-18-076; ABC-18-19). A written informed consent for enrollment or consent to continue and use patient data was obtained from each patient or their legal surrogate. The protocol was registered (TRIAL REGISTRATION: ClinicalTrials.gov Identifier: NCT 03557229).

### 2.2. Randomization, Masking, and Drug Administration

**Figure 1:**
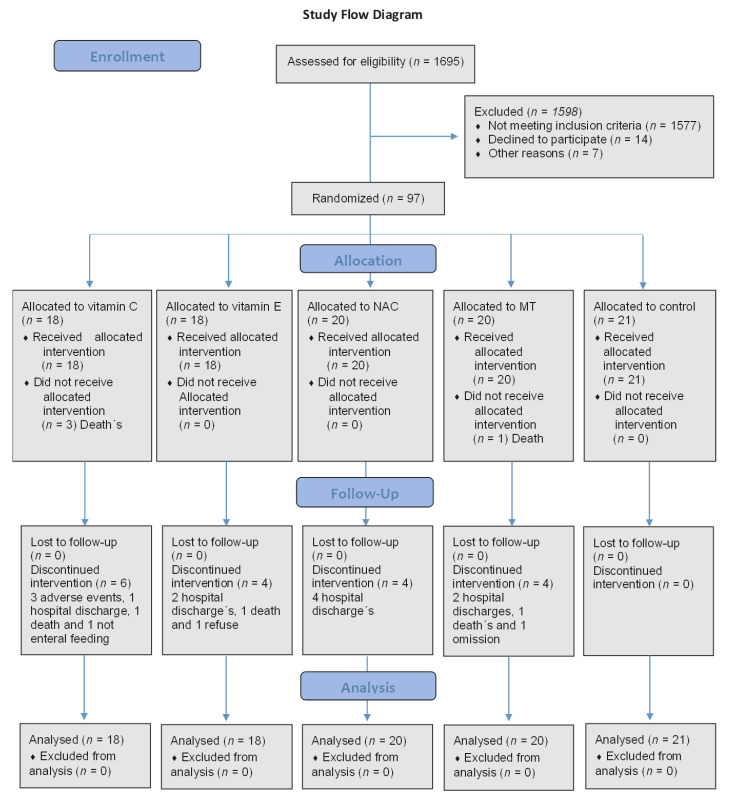
Flow diagram of the study. Abbreviations: Vit C = vitamin C, Vit E = vitamin E, NAC = n-acetylcysteine, MT = melatonin.

A total of five treatments were used each in an independent group of 18 patients. Group 1 received Vit C, group 2 Vit E, group 3 NAC, group 4 MT and group 5 control (this group did not receive any type of antioxidant therapy).The control group did not receive treatment since the treating physician did not agree for the patient to receive any antioxidant. However, the patients agreed that samples could be processed. All antioxidants were administered orally or through a nasogastric tube during 5 days in addition to the standard therapy. The random allocation sequence for the administration of the antioxidants was generated at the coordinating center, using a computer-generated random program (Figure 1). Blinding was maintained by the investigational pharmacy at each institution. Researchers were also blinded from the onset of the study until the analysis of the outcomes.

All antioxidants were orally administered or applied through a nasogastric tube for 5 days. Tablets of 600 mg every 12 h of NAC were used. Further, 50 mg of MT in capsules of 5 mg were given to patients once a day, and 1 mg Vit C tablets were administered every 6 h. Vit E capsules of 400 UI were given every 8 h. The doses of antioxidants were chosen according to what has been reported in the literature [24,25,26,27]. All data entry was monitored at the coordinating center, with site visits for source data verification. Also, patients were equally distributed, and all patients were analyzed. For patients receiving Vit C (*n* = 18), there were 3 deaths. For patients receiving Vit E (*n* = 18) there were 3 deaths. For patients receiving NAC (*n* = 20), there were 2 deaths, and for those receiving MT (*n* = 20), there were 4 deaths. Finally, in the control group (*n* = 21), there were 5 deaths.

### 2.3. Standard Therapy at the ICU

Patients were treated according to the recommendation of the International Guidelines for Management of Sepsis and Septic Shock. For the evaluation of the outcome, the SOFA scores for 5 days were the primary result. Additionally, 14 pre-specified laboratory results were determined, including plasma OS markers such as nitrate/nitrite (NO_3_^−^/NO_2_^−^) ratio, LPO, glutathione (GSH) levels, total antioxidant capacity (TAC), carbonylation and Vit C levels at 48 h. Other secondary outcomes were measured on day 28 including mortality due to any cause, ventilator-free days, ICU-free days, and hospital-free days. Ventilator-free days were defined as the number of days a patient was extubated from mechanical ventilation, after ICU admission. When reintubation was required the days without intubation were subtracted from the total days. If the patient died in the hospital, a value of zero was assigned to post-extubation. ICU-free days began the moment the patient was transferred out of the ICU to day 28. Hospital- and ICU-free days were calculated similarly.

### 2.4. Study Measurements and Procedures

To evaluate the organ dysfunction, the SOFA score (neurologic, respiratory, hemodynamic, hepatic, and hematologic) was calculated on admission and during the days of treatment. The CRP and the PCT determinations were performed on admission, before the beginning of the antioxidant therapy, and during the next 7 days.

### 2.5. Sampling for the Determination of Oxidative Stress and Antioxidant State

The measurement of OS markers was done before the beginning of the antioxidant therapy and 48 h after its initiation.

### 2.6. Sample Obtainment and Storage

Blood samples were obtained from each patient that entered the draw, before initiation of the treatment and 48 h after its administration. The blood samples were centrifuged for 20 min at 936× *g* and 4 °C. The plasma of the samples was placed in 3 or 4 aliquots and stored at −30 °C.

### 2.7. Oxidative Stress Markers in Plasma

#### 2.7.1. NO_3_^−^/NO_2_^−^ Ratio

The NO_3_^−^ was reduced to NO_2_^−^ by the nitrate reductase enzyme reaction. 100 μL of plasma previously deproteinization with 0.5 N, NaOH and 10%, ZnSO_4_ were mixed and the supernatant was incubated for 30 min at 37 °C in presence of nitrate reductase (5 units). At the end of the incubation period, 200 µL of sulfanilamide 1% and 200 µL of N-naphthyl-ethyldiamine 0.1% were added and the total volume was adjusted to 1 mL. The absorbance was measured at 540 nm [28].

#### 2.7.2. LPO Levels

Briefly, 50 µL CH3-OH with 4% butylated hydroxytoluene plus phosphate buffer pH 7.4 was added to 100 µL of plasma. It was incubated and centrifuged at 4000 rpm in room temperature for 2 min. Then, the n-butanol phase was extracted, and absorbance was measured at 532 nm [28].

#### 2.7.3. GSH Concentration

Briefly, 800 μL of phosphate buffer 50 mM, pH 7.3, plus 100 μL of Ellman reactive (5,5′ dithiobis 2-nitrobenzoic) 1M were added to 100 μL of plasma prior to deproteinization with 20% trichloroacetic acid (*v*/*v*). It was incubated at room temperature and absorbance was read at 412 nm [28].

#### 2.7.4. Evaluation of TAC

Briefly, 100 μL of plasma were suspended in 1.5 mL of a reaction mixture prepared as follows: 300 mM acetate buffer pH 3.6, 20 mM hexahydrate of ferric chloride, and 10 mM of 2,4,6-Tris-2- pyridil-s-triazine dissolved in 40 mM HCl. These reactive were added in a relation of 10:1:1 *v*/*v*, respectively. After mixing, samples were incubated at 37 °C for 15 min in the dark. The absorbance was measured at 593 nm [28].

#### 2.7.5. Carbonylation Protein Concentration

Briefly, 100 μL of plasma were added to 500 μL of HCl 2.5 N in parallel with another sample with 500 μL of 2, 4-dinitrophenylhydrazine (DNPH) and incubated. At the end of the incubation period, they were centrifuged at 15,000× *g* for 5 min. The supernatant was discarded. Two washings were performed. The mixture was incubated again at 37 °C for 30 min. Absorbance was read in a spectrophotometer at 370 nm, using bi-distilled water as blank and a molar absorption coefficient of 22,000 M^−1^ cm^−1^ [28].

#### 2.7.6. Vitamin C Levels

Briefly, 100 μL of 20% trichloroacetic acid were added to 100 μL of plasma and centrifuged at 5000 rpm for 5 min. Then, 200 μL of Folin-Ciocalteu reagent 0.20 mM was added to the supernatant. The mixture was incubated for 10 min. The absorbance was measured at 760 nm [28].

### 2.8. Statistical Analysis

Based on a SD of 2.9 of the SOFA score, the study was estimated to require 55 patients (11 per group) to have 84% power (2-sided with an α = 0.05) and 160 (32 per group) for 100% power. In accordance with these calculations, our study enrolled 97 patients to allow for a 10% of dropouts, providing a statistical power of 99%, with an α = 0.05. Testing was 2-sided. Effects are reported with a point estimate and 95% CIs in addition to *p* values.

Group comparisons were made using χ^2^ tests for equal proportions, *t* tests for normally distributed data, Kruskal–Wallis and Wilcoxon rank sum tests otherwise, with results presented as frequencies with percentages, means with SDs, and medians with minimum and maximum, respectively.

The primary end point of the SOFA score and the secondary end points CRP and PCT were analyzed with a mixed linear model and fit to repeated-measures analysis of variance. The model included 1 between-participant factor (group (Vit C, Vit E, NAC, MT, no treatment [control])), 1 within-participant factor (time (0, 1, 2, 3, 4, and 5 days)), and the interaction between group and time, testing the hypothesis that differences between treatment groups are the same over time. Because of a potential for type I error caused by multiple comparisons, findings for analyses of secondary end points should be interpreted as exploratory. Statistical analysis was performed with Stata version 15.1.

## 3. Results

### 3.1. Characteristics of the Patients

From July 2018 to November 2019 a total of 1695 eligible patients were identified, of whom 1598 were excluded (reasons listed in Figure 1). Ninety-seven patients were randomized, with 18 assigned to each antioxidant and 21 to the control group. Of all patients included, none was lost in the follow up. Baseline demographic data (age, gender, etc.) were similar between the groups (Table 1).

### 3.2. Treatments

Treatments were given for a median of five days. The median of adherence in the 4 different groups of treatment was 100%. There was no difference between groups in the time from meeting eligibility criteria to the first dose, the time receiving the treatment, and the adherence.

#### Primary Outcome

**Figure 2:**
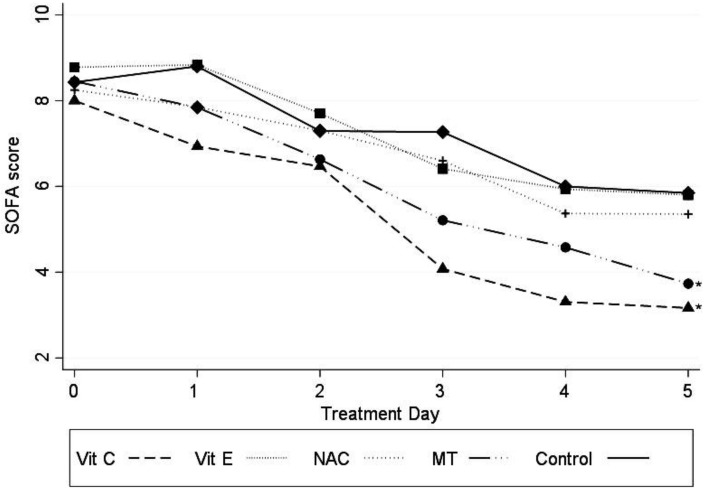
SOFA score variations in patients with the different antioxidant treatments. Abbreviations: SOFA=sequential organ failure assessment, Vit C = vitamin C, Vit E = vitamin E, NAC = n-acetylcysteíne, MT = melatonin, Const = constant. Marginal approximation model considering the control group as a base: Vit C −1.94 (−2.95 to −0.94; *p* < 0.001); Vit E −0.14 (−1.10 to 0.81; *p* = 0.77); NAC −0.62 (−1.55 to 0.30; *p* = 0.18); MT −1.27 (−2.21 to −0.34; *p* = 0.007); Const 7.46 (6.78 to 8.13). * *p* ≤ 0.007.

Patients receiving MT and Vit C showed a significant decrease in SOFA score (−1.27 (95% CI −2.21 to −0.34); *p* = 0.007 for MT and −1.94 (95% CI −2.95 to −0.93); *p* < 0.001 for Vit C) (Figure 2).

The LPO levels were significantly reduced in patients treated with MT (*p* = 0.04) and there was a significant decrease in NO_3_^−^/NO_2_^−^ levels in patients with lung infection treated with Vit C (*p* < 0.01), Table 2.

**Figure 3:**
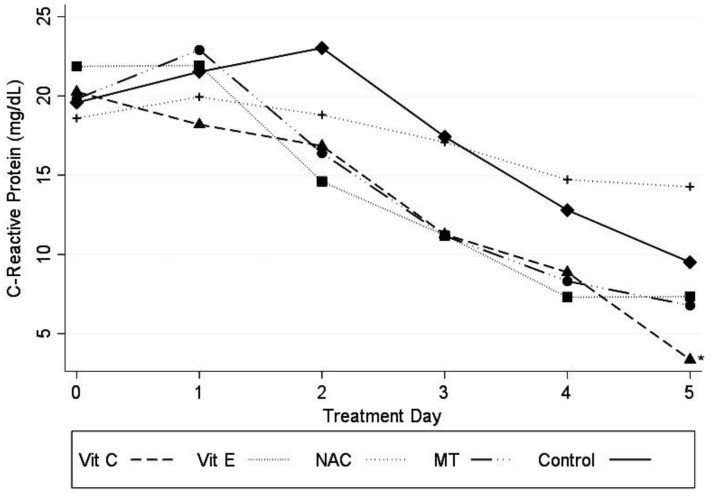
Variations in CRP levels in plasma of patients in receiving the different antioxidant treatments. Abbreviations: CRP = C-reactive protein, Vit C = vitamin C, Vit E = vitamin E, NAC: n-acetylcysteíne, MT = melatonin, Cons = constant. Marginal approximation model considering the control group as a base: Vit C −3.82 (−7.49 to −0.15; *p* ≤ 0.05); Vit E −2.97 (−6.54 to 6.01; *p* = 0.103); NAC −2.41 (−3.74 to 3.25; *p* = 0.892); MT −2.30 (−5.88 to 1.27; *p* = 0.207); Cons 17.9 (15.45 to 20.36) * *p* ≤ 0.05.

Patients receiving Vit C had a significant decrease in CRP levels on the different days of treatment (*p* ≤ 0.05), as shown in Figure 3.

**Figure 4:**
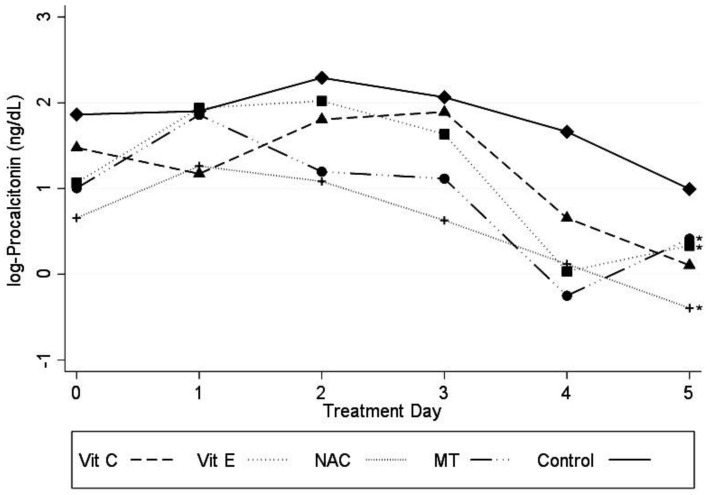
Log PCT concentration in the plasma from patients of the experimental groups with the different antioxidant treatments. Abbreviations: Log PCT = logarithm of procalcitonin, Vit C = vitamin C, Vit E = vitamin E, NAC = n-acetylcysteíne, MT = melatonin. Marginal approximation model considering the control group as a base: Vit C −0.43 (−1.03 to 0.15; *p* = 0.149); Vit E −0.59 (−1.18 a −0.006; *p* ≤ 0.05); NAC −0.92 (−1.48 to −0.35; *p* = 0.001); MT −0.57 (−1.15 to 0.006; *p* = 0.05); Cons 1.46 (1.05 to 1.86); * *p* ≤ 0.05.

PCT levels were significantly decreased in patients receiving Vit E, NAC, and MT (*p* < 0.05), as shown in Figure 4. Carbonylation levels tended to be reduced before treatment and Vit E tended to decrease its level after treatment (*p* = 0.07) without there being a statistically significant difference.

Regarding the secondary outcomes, 13 patients (13.68%) required renal replacement therapy, 63 (65.63%) needed mechanical ventilation and 17 (17.89%) died. There was no statistically significant difference in days free of renal replacement therapy, mechanical ventilation, ICU stay length, or hospitalization at 28 days. There was also no statistically significant difference in intrahospital mortality.

### 3.3. Undesired Side Effects

A patient receiving Vit C presented abdominal pain and another patient underwent a skin rash. Only one patient who received MT reported drowsiness. No adverse events were reported in patients with NAC or Vit E.

## 4. Discussion

Treatment with antioxidants as an adjuvant in the standard management of patients with sepsis and/or septic shock has been suggested [29,30]. We studied critically ill patients with septic shock, regardless of the etiology and site of infection. All patients had initial low levels of Vit C. This was related with the severity of organ failure and mortality [17]. The decrease in Vit C levels confirms the reported hypovitaminosis (<0.23 µM ascorbic ac/mL) in septic shock [31]. This condition may be due to augmented metabolic demand since intestinal absorption was not compromised in the patients in our study [32]. Vit C restored the normal values of this vitamin, and organ function was improved. The best result was found in subjects with pneumonia which showed a statistically significant difference. This finding is in agreement with previous results [33,34]. The combined use of Vit C, thiamine, and steroids has recently been suggested. It is still necessary to compare if the use of Vit C alone has worse effects than the combinations [35]. In patients with septic shock, the administration of Vit C and MT improved the organ dysfunction assessed by the SOFA score. This finding could be associated to a decrease in the NO_3_^−^/NO_2_^−^ ratio and LPO levels.

The Vitamin C infusion for treatment in sepsis induced acute lung injury (CITRIS-ALI) study in patients with acute respiratory distress syndrome, and organ failure showed no improvement with Vit C [36]. The median time before starting treatment with Vit C was of 5 h in this study, and markers such as CRP were significantly decreased, as in another previous study [37]. The possible difference between the findings of this study and our results could be related to the fact that, in the CITRIS-ALI study, they started the therapy with Vit C later than we did.

The VITAMINS trial showed no significant difference in the SOFA score, or in days without ventilation. However, the use of Vit C lowered mortality [38]. In that same study, CRP levels were not decreased, which was probably due to the late administration of Vit C in advanced stages of sepsis before developing acute respiratory distress syndrome (ARDS) [37]. In contrast, we found a decrease in the levels of NO_3_^−^/NO_2_^−^ which is relevant, since Vit C inhibits the production of superoxide and peroxynitrite, thus preventing abundant NO synthesis, inhibiting mRNA expression and decreasing pathological vasoconstriction [16]. These effects might underlie the clinical benefits of the treatment. A shorter time of use of vasopressors and decreased intrahospital mortality was found in patient receiving Vit C [39].

This is the first study in which the use of MT has been tested in humans with septic shock. Recently MT has been applied in subjects with COVID 19 and it had a high safety profile limiting the disease. Experimental and clinical studies are required to confirm this hypothesis [40]. MT possesses free radical scavenging properties thus protecting cell membrane lipids, cytosol proteins, and nuclear and mitochondrial DNA [29]. In our findings, LPO was significantly decreased in the group of patients who received MT. This result resembled the findings in Galley’s study [29]. MT has beneficial effects in experimental cells, plants, and animals. However, its mechanisms of action remain unknown. The effects of MT might be related to its detoxifying ability, thus protecting molecules from the destructive effects of OS in ischemia/reperfusion (stroke, heart attack), ionizing radiation and drug toxicity. In sepsis, the protective effects of MT are associated with the inhibition of the apoptotic processes and the reduction of OS [41].

Production of ROS was increased in an animal model of septic shock [42]. This coincides with a lowering of the TAC and a reduction of the activity of superoxide dismutase and GSH peroxidase [43]. MT reversed morphological damage and increased the activities of antioxidant enzymes [44,45,46]. Therefore, research through blinded clinical trials and multicenter studies with adequate amounts of MT are needed to determine the potential of MT as an antioxidant treatment [47]. In this clinical trial, we found a reduction of LPO and a potentially benefic effect of MT in organ dysfunction. Its use as an adjuvant in septic shock reduces inflammation and oxidation in animal models with respiratory damage induced by infection. MT has positive physiological actions and could be effective and safe for patients with septic shock of any etiology, including those infected with SARS-CoV-2 [7].

The use of NAC improved the antioxidant capacity and tended to increase GSH, although the difference was not statistically significant. This confirms its antioxidant effect through the replacement of GSH deposits [12]. NAC decreased organ failure, confirming previous findings [14]. Other antioxidants such as polyphenols, β-glucan, and antioxidants targeting mitochondria, selenium salts, and selenium organ compounds are effective for improving OS in sepsis. The study of their pathophysiological implications justifies the combined therapy with antioxidants and standard treatments.

Vit E tended to decrease LPO and carbonylation. This vitamin protects cell membranes from LPO, ending the chain reaction. It is also an O_2_^−^ and OH sequestrant [48].

**Figure 5:**
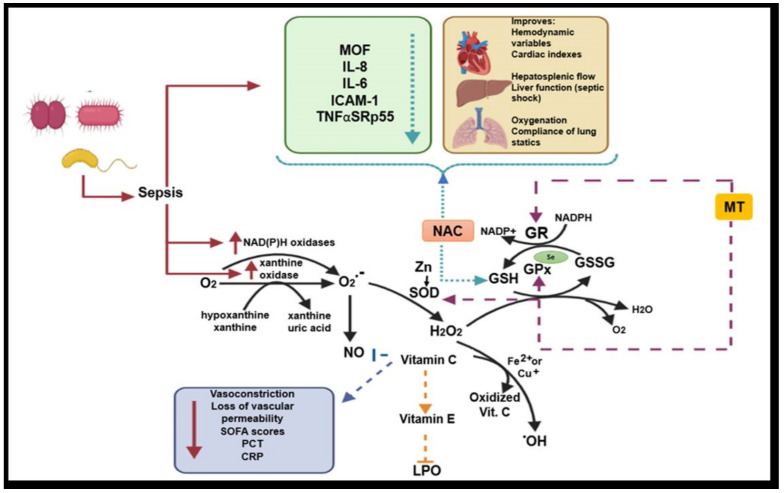
Role of antioxidant systems and enzyme cofactors in the management sepsis and ROS formation. Abbreviations: O_2_^−^ = superoxide anion, NO = nitric oxide, Zn = zinc, SOD = superoxide dismutase, H_2_O_2_ = hydrogen peroxide, OH = hydroxyl radical, NAC = N-acetylcysteine, MT = melatonin, GSH = glutathione, GSSG = oxidized glutathione, GR = glutathione reductase, GPx = glutathione peroxidase, TNFαSRp55 = soluble α receptor tumor necrosis factor p55, MOF = multiple organ failure, LPO = lipoperoxidation, CRP = C reactive protein, SOFA = sequential organ failure assessment.

In summary, antioxidants benefit subjects with septic shock. Septic shock is triggered by bacterial stimuli, fungi, or viruses. In this medical condition, it is necessary to regulate inflammation and other mechanisms that lead to OS [48]. In Figure 5, we show the mechanisms involved in the oxidative stress mismatch, the role they play during the induction of damage, and we describe the role of antioxidant systems and enzyme cofactors in the management of sepsis.

## 5. Conclusions

Adding antioxidants to standard therapy regulates inflammation in patients with septic shock. In pulmonary sepsis, replacement therapy with Vit C increases its serum levels, and decreases the levels of CRP, PCT, and NO_3_^−^/NO_2_^−^. MT decreases LPO and the SOFA score. NAC reduces LPO and improves the antioxidant capacity. Vit E tends to decrease LPO. Each antioxidant has beneficial effect. Thus, they might be combined in clinical trials in patients with septic shock.

## 6. Limitations

The absorption may be altered by the enteral route of administration. However, we found increases of Vit C levels in serum. The present trial is underpowered to detect differences in mortality and in outcomes between groups because the sample size was calculated for differences in OS.

## Figures and Tables

**Table 1:** General characteristics of the patients during hospital stay.

Characteristics	Vit C (*n* = 18)	Vit E (*n* = 18)	NAC (*n* = 20)	MT (*n* = 20)	C (*n* = 21)
Age (median, min–max)	62 (22–95)	65.5 (22–91)	67.5 (18–95)	62.5 (46–95)	76 (51–89)
Weight kg (median, min–max)	71 (33–112)	71.5 (40–120)	69.5 (39–95)	67 (50–106)	68 (50–105)
BM weight/height^2^ (median, min–max)	25.4 (14.7–40.4)	25 (15.1–41.4)	22.45 (16.5–0.3)	25.35 (17.3–52)	25.4 (19.6–58)
Gender (%)					
Men	6 (6.19)	12 (12.37)	11 (11.34)	10 (10.31)	10 (10.31)
Women	12 (12.37)	6 (6.19)	9 (9.28)	10 (10.31)	11 (11.34)
Chronic health condition (%)					
Diabetes Mellitus	4 (4.12)	4 (4.12)	3 (3.09)	5 (5.15)	6 (6.19)
Hypertension	6 (6.19)	8 (8.25)	9 (9.28)	7 (7.22)	11 (11.34)
Cancer	5 (5.15)	9 (9.28)	7 (7.22)	7 (7.22)	11 (11.34)
Chronic renal failure	1 (1.03)	2 (2.03)	4 (4.12)	3 (3.09)	2 (2.06)
Admission source (%)					
Emergency department	9 (9.28)	12 (13.37)	10 (10.31)	14 (14.43)	9 (9.28)
Operating room	4 (4.12)	2 (2.06)	3 (3.09)	2 (2.06)	4 (4.12)
Inpatient ward transfer	3 (3.09)	4 (4.12)	7 (7.22)	4 (4.12)	7 (7.22)
Other	2 (2.06)	0	0	0	1 (1.03)
Primary site of infection (%)					
Pulmonary	7 (7.37)	9 (9.97)	9 (9.97)	8 (8.42)	6 (6.32)
Gastrointestinal	7 (7.37)	3 (3.16)	4 (4.21)	3 (3.16)	9 (9.97)
Urinary	2 (2.11)	2 (2.11)	5 (5.26)	5 (5.26)	3 (3.16)
CNS	0	2 (2.11)	0	0	1 (1.05)
Blood	0	1 (1.05)	0	2 (2.11)	0
Physiological variables 24 h					
before randomization					
(median, min–max)					
White blood cell count × 10^3^/μL	11 (5.1–39.9)	10.8 (0.4–25.4)	8.6 (0–32.5)	11.7 (5.2–29.6)	12 (0.9–49.8)
Platelet count × 10^3^/μL	256 (7–409)	158 (10–363)	155 (22–470)	187.5 (29–543)	225 (24–436)
Lactate (mmol/L)	1.65 (0–4.8)	2.1 (0.82–10.5)	1.74 (0.99–7.8)	2.27 (1–17)	2.52 (1.1–12.4)
Serum creatinine (mg/dL)	0.9 (0.5–5.5)	1.35 (0.5–3.8)	0.92 (0.5–6.6)	1.27 (0.57–6.6)	1.2 (0.5–5.2)
Bilirubin (mg/dL)	0.75 (0.23–3.5)	1.05 (0.35–4.4)	0.80 (0.2–4)	1.03 (0.17–3.7)	1.15 (0.2–13.6)
PaO_2_/FiO_2_ (mmHg)	168.5 (61–408)	215 (39–271)	146 (71–367)	197 (57–261)	197 (131–560)
C reactive protein (mg/dL)	18.33 (1.9–1.4)	20.12 (0.5–47)	13.34 (0.02–6.7)	21.75 (1.35–6.7)	20.25 (1.36–5.3)
Procalcitonin (ng/dL)	1.46 (0.16–321)	2.92 (0.08–109)	2.35 (0.06–95.5)	2.32 (0.22–38.7)	8.25 (0.08–100)
Intervention before randomization (%)					
Mechanical ventilation	11 (11.58)	9 (9.47)	14 (14.47)	12 (12.63)	16 (16.84)
Vasopressors	9 (9.38)	7 (7.29)	12 (12.50)	9 (9.38)	11 (11.46)
Norepinephrine	0	1 (1.04)	0	0	0
Vasopressin	8 (8.33)	10 (10.42)	8 (8.33)	11 (11.46)	10 (10.42)
Norepinephrine plus vasopressin					
Inotropes					
Dobutamine	0	0	0	0	1 (1.04)
Levosimendan	0	5 (5.21)	1 (1.04)	3 (3.13)	5 (5.21)
Dopamine	1 (1.04)	0	0	1 (1.04)	0
Renal replacement Therapy	1 (1.04)	2 (2.08)	2 (2.08)	1 (1.04)	3 (3.13)
Corticosteroid use before					
randomization during the study (%)	6 (6.19)	11 (11.34)	9 (9.28)	8 (8.25)	10 (10.31)
SAPS II (median, min–max)	38 (16–62)	40 (24–73)	38.5 (12–97)	41.5 (13–73)	40 (18–79)
APACHE III (median, min–max)	13.5 (5–47)	19 (11–33)	14.5 (5–46)	17 (6–39)	15 (5–38)
SOFA score (median, min–max)	8.5 (3–16)	8.5 (5–14)	8.5 (1–17)	8 (3–14)	8 (1–16)
Time from ICU admission to randomization hours (median, min–max)	5 (1.5–70)	6 (1–17)	3 (1–140)	9 (3–48)	-

The data presented in this table are on admission to intensive care. Several patients met the inclusion criteria several hours or days after admission to the intensive care unit and all patients used vasopressors since it was an inclusion criterion. Abbreviations: Vit C: vitamin C; Vit E: vitamin E; NAC: n-acetylcysteine; MT: melatonin; (min-mx): minimum–maximum; BMI: body mass index; CNS: central nervous system; SAPS II: Simplified Acute Physiology Score; APACHE, Acute Physiology and Chronic Health Evaluation; SOFA, Sequential Organ Failure Assessment; ICU: intensive care unit.

**Table 2:** Oxidative stress markers before and after 48 h of antioxidant therapy.

Lipid Peroxidation (nM MDA/mL of Plasma)			
	Pre	Post	*p*
Vit C (*n* = 18)	3.44 (0.52–19.62)	2.81 (0.23–8.70)	0.14
Vit E (*n* = 18)	4.33 (1.25–15.25)	3.24 (0.38–12.07)	0.17
NAC (*n* = 20)	3.46 (0.23–9.49)	3.46 (0.38–11.01)	0.77
MT (*n* = 20)	2.13 (0.23–11.68)	2.42 (0.23–7.11)	0.04
Control (*n* = 21)	3.44 (0.52–9.49)	3.90 (0.23–9.10)	0.75
**NO_3_ + NO_2_** **(µM/mL of plasma)**			
	Pre	Post	*p*
Vit C (*n* = 18)	2.10 (0.98–2.73)	1.49 (0.03–2.57)	<0.01
Vit E (*n* = 18)	1.79 (0.53–3.81)	2.00 (0.76–5.65)	0.36
NAC (*n* = 20)	2.43 (0.80–7.02)	2.15 (0.01–8.16)	0.81
MT (*n* = 20)	1.72 (0.67–4.77)	1.32 (0.03–7.42)	0.19
Control (*n* = 21)	2.25 (0.28–2.76)	2.24 (0.01–7.22)	0.97
**Total antioxidant capacity** **(nM/mL of plasma)**			
	Pre	Post	*p*
Vit C (*n* = 18)	2226.2 (747.6–3053.4)	2050.9 (966.6–2551.8)	0.11
Vit E (*n* = 18)	2148.4 (886.3–3287.6)	2223.1 (618.3–3841.9)	0.90
NAC (*n* = 20)	1453.6 (621.5–2351.4)	1951 (812.6–3528.7)	0.05
MT (*n* = 20)	1999 (561.3–2519.2)	1747.5 (456.5–2745.6)	0.59
Control (*n* = 21)	2451.6 (1600–3467.1)	2064.7 (312.4–3501)	0.42
**Carbonylation** **(ng/mL of plasma)**			
	Pre	Post	*p*
Vit C (*n* = 18)	48.85 (10.90–114.53)	44.76 (12.72–98.17)	0.59
Vit E (*n* = 18)	52.26 (27.27–137.25)	42.723 (21.36–89.53)	0.07
NAC (*n* = 20)	40.22 (22.27–89.99)	41.13 (22.72–93.17)	0.47
MT (*n* = 20)	74.76 (8.63–181.34)	62.721 (29.99–142.25)	0.40
Control (*n* = 21)	46.359 (9.99–106.80)	44.08 (26.36–111.80)	0.28
**GSH concentration** **(nM/mL of plasma)**			
	Pre	Post	*p*
Vit C (*n* = 18)	0.10 (0.01–0.24)	0.08 (0.01–0.20)	0.50
Vit E (*n* = 18)	0.05 (0.00–0.30)	0.07 (0.00–0.32)	0.38
NAC (*n* = 20)	0.08 (0.00–0.54)	0.10 (0.009–0.57)	0.14
MT (*n* = 20)	0.07 (0.00–0.32)	0.07 (0.010–0.51)	0.64
Control (*n* = 21)	0.06 (0.03–0.20)	0.05 (0.01–0.16)	0.15
**Vit C** **(µM/mL of plasma)**			
	Pre	Post	*p*
Vit C (*n* = 18)	0.17 (0.04–0.87)	0.27 (0.06–0.99)	<0.01
Vit E (*n* = 18)	0.27 (0.08–0.99)	0.26 (0.12–0.79)	0.58
NAC (*n* = 20)	0.21 (0.09–0.61)	0.18 (0.00–0.96)	1.00
MT (*n* = 20)	0.21 (0.04–0.56)	0.21 (0.04–0.43)	0.83
Control (*n* = 21)	0.22 (0.08–0.77)	0.19 (0.07–0.64)	0.02

Abbreviations: Pre: pre-treatment; Post: post-treatment; Vit C: vitamin C; Vit E: vitamin E; NAC: n-acetylcysteine; MT: melatonin. All values are expressed as median (minimum-maximum). Wilcoxon matched pairs signed rank tests. The bold in the table is to highlight the results with statistical change.
